# Adding a toe joint to a prosthesis: walking biomechanics, energetics, and preference of individuals with unilateral below-knee limb loss

**DOI:** 10.1038/s41598-021-81565-1

**Published:** 2021-01-21

**Authors:** Kirsty A. McDonald, Rachel H. Teater, Justin P. Cruz, John T. Kerr, Gerasimos Bastas, Karl E. Zelik

**Affiliations:** 1 Department of Mechanical Engineering, Vanderbilt University, Nashville, TN 37212 USA; 2 Department of Exercise Physiology, University of New South Wales, Sydney, NSW 2052 Australia; 3 Department of Biomedical Engineering, Vanderbilt University, Nashville, TN 37212 USA; 4 Department of Physical Medicine and Rehabilitation, Vanderbilt University, Nashville, TN 37212 USA

**Keywords:** Translational research, Musculoskeletal system

## Abstract

Toe joints play an important functional role in able-bodied walking; however, for prosthesis users, the effect of adding a toe joint to a passive prosthetic foot remains largely unknown. The current study explores the kinematics, kinetics, rate of oxygen consumption and user preference of nine individuals with below-knee limb loss. Participants walked on a passive prosthetic foot in two configurations: with a Flexible, articulating toe joint and with a Locked-out toe joint. During level treadmill gait, participants exhibited a decrease in Push-Off work when using the Flexible toe joint prosthesis versus the Locked toe joint prosthesis: 16% less from the prosthesis (p = 0.004) and 10% less at the center of mass level (p = 0.039). However, between configurations, participants exhibited little change in other gait kinematics or kinetics, and no apparent or consistent difference in the rate of oxygen consumption (p = 0.097). None of the traditional biomechanical or metabolic outcomes seemed to explain user preference. However, an unexpected and intriguing observation was that all participants who wore the prosthesis on their dominant limb preferred the Flexible toe joint, and every other participant preferred the Locked configuration. Although perhaps coincidental, such findings may suggest a potential link between user preference and limb dominance, offering an interesting avenue for future research.

## Introduction

In humans, the metatarsophalangeal (toe) joints extend and flex during walking. This toe joint articulation affects musculoskeletal dynamics within the foot and ankle^1–3^, as well as whole-body gait biomechanics^4^. Both theoretical and experimental findings indicate that altering or augmenting toe joint articulation dynamics can impact key variables related to gait economy^5,6^ and stability^7,8^.

**Figure 1:**
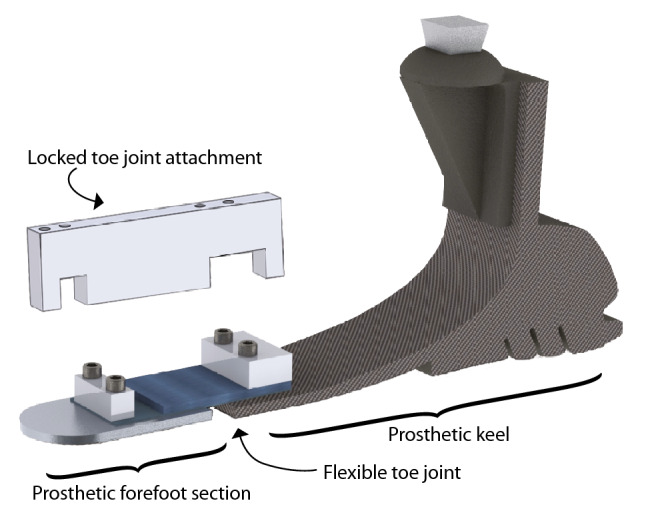
A custom-modified Össur Balance Foot J passive prosthesis shown in a Flexible toe joint configuration. The aluminum block attachment (above) can be secured over the joint to create a Locked-out toe joint configuration.

Recent work from our laboratory found that changing toe joint stiffness has a sizeable effect on center of mass Push-Off power during walking, to an extent comparable with changing ankle joint stiffness^4^. Here, the authors utilized adapted walking boots to immobilize the biological ankle joints of participants whilst enabling the attachment of prosthetic feet to each boot base. In the case of prosthetic devices, a toe joint refers to the articulating region that connects the prosthetic keel and the section equivalent to the biological forefoot (e.g., Fig. 1). Interestingly, in this study nine of the ten participants reported that they preferred walking on a foot prosthesis with an articulating toe joint versus one without a toe joint. Furthermore, a small sample of prostheses with toe joints have recently entered the commercial market (e.g., Ottobock Meridium, ST&G ToeFlex). Whether this feature is preferable and/or beneficial relative to a fully rigid/stiff keel, and how this may vary according to locomotor task, remains unclear. Together, these prior research findings and contemporary commercial devices motivated us to explore the effects and implications of toe joint dynamics on lower limb prosthesis users, most of whom walk on commercially-available prosthetic feet that do not include an articulating toe joint.

Prosthesis design and prescription related to toe joint articulation could benefit from multi-subject studies, assessing the effect of toe joint dynamics on the biomechanics and energetics of lower limb prosthesis users during walking. Previously, Zhu and colleagues^9^ assessed the effect of adding a toe joint to the foot keel of a powered prosthesis during walking, but only with a single prosthesis user. The authors observed improved ground reaction force symmetry, which they attributed to restoring toe joint articulation. Yet it remains unclear whether prosthesis users subjectively prefer to have a toe joint or not during ambulation. It is also noteworthy to add that cosmetic foot shells typically include aesthetic toes (made of rubber or foam) which, in combination with shoes, often extend out beyond the prosthetic foot keel. However, it also remains unknown whether these aesthetic toes behave functionally as a toe joint, or if the stiffness properties of the cosmesis provide auxiliary benefits during walking and other locomotor tasks.

To address these knowledge gaps, the objective of this study was to compare the biomechanics, energetics, and user preference of individuals with unilateral below-knee limb loss walking on a prosthetic foot with and without a toe joint. Based on the findings of Honert and colleagues^4^, it was hypothesized that the addition of a toe joint would reduce center of mass Push-Off work.

## Methods

### Participants

Healthy, active persons with unilateral below-knee limb loss (*N* = 9; Medicare Functional Classification Level: K3/K4; age: 41 ± 11 years; body mass: 94 ± 13 kg; height: 1.84 ± 0.05 m; mean ± standard deviation) provided their informed written consent before participating in this study, which was approved by the Institutional Review Board at Vanderbilt University. Sample size calculations were originally computed based on peak center of mass Push-Off power (and work). Early pilot data had suggested differences on the order of 30–45 W, and previous studies on hemiparetic^10,11^ and elderly gait^12^ had interpreted similar Push-Off differences to be clinically meaningful. Using this mean difference range and standard deviation of 30 W, and assuming alpha = 0.05 and power = 0.8, paired t-test sample size calculations indicated a need for 7–16 participants. Our study on nine participants was on the lower end of this range and does not guarantee that the study was sufficiently powered for all other (non-Push-Off) outcomes.

Participants capable of normal community ambulation were required for this study, given the physical demands of this four-day, multi-task protocol. Three foot prostheses were modified and available to accommodate body masses from 75 to 120 kg and shoe sizes from US men’s 7–12. Individuals were excluded from this study if they did not fit these size requirements. Individual participant demographics are presented in Table 1.

**Table 1:** Participant demographics.

Participant ID	Age (years)	Body mass (kg)	Height (m)	Leg length (m)	Years since limb loss	K-level	Cause of limb loss	Daily-use prosthesis
1	33	119.1	1.92	1.00	3.8	4	Traumatic	Fillauer Formula
2	49	94.4	1.86	0.95	7.5	4	Traumatic	Fillauer AllPro
3	33	97.8	1.88	0.98	9.3	4	Traumatic	Össur Pro-Flex XC
4	55	100.5	1.83	0.94	4.7	3	Vascular	Fillauer AllPro
5	28	81.5	1.90	0.99	8.8	4	Traumatic	Fillauer AllPro
6	41	99.7	1.80	0.93	41.0	4	Congenital	Ottobock Triton
7	47	83.9	1.76	0.97	7.4	4	Traumatic	Fillauer AllPro
8	52	73.4	1.85	0.98	2.5	4	Traumatic	Fillauer Formula
9	28	97.4	1.78	0.94	5.3	4	Traumatic	Fillauer AllPro
Mean ± SD	40.7 ± 10.5	94.1 ± 13.3	1.84 ± 0.05	0.96 ± 0.02	10.0 ± 11.8			

### Experimental prosthesis

We modified three categories of a single commercial prosthesis (Balance Foot J, Össur, Categories 25, 27 and 28) such that each could function in two configurations: (i) with a Flexible toe joint, which was accomplished by using sheets of spring steel affixed between the foot keel and toe segment, and (ii) with a Locked toe joint which used an aluminum block to prevent flexion and extension, thus effectively creating a solid foot keel without a toe joint (Fig. 1). The Flexible toe joint was designed to have a stiffness of 0.34 Nm degree^−1^, an intermediate stiffness selected based on results of Honert and colleagues^4^. The prosthesis was housed in a modified cosmetic foot shell only (i.e., no shoe was used) to allow foot and toe joint markers to be visible and to avoid confounds due to the bending stiffness of the shoe itself. When communicating with participants during training and testing, we simply referred to these as Foot One and Foot Two, to minimize the risk of biasing participant preferences.

### Protocol

The study protocol described below was developed in conjunction with institutional guidelines. It involved four sessions: two training and two testing. Training sessions were separated by at least 24 h and, at most, 11 days. No more than 7 days elapsed from the final training to the first testing session. Testing sessions were separated by 24 h when possible; however, two participants required both testing sessions to be conducted on the same day due to availability constraints. The acclimation protocol described below was chosen based on pilot testing and our experience with prior prosthetics studies^4,13,14^.

### Training sessions

At the beginning of the first session, participants wore their prescribed prosthesis and were provided time to familiarize themselves with several locomotor tasks and laboratory equipment. The analysis and results detailed in this manuscript are only on level walking, however this was part of a larger protocol which also included stair ascent/descent, ramp ascent/descent and walking over uneven terrain.

Next, the experimental prosthesis was fit and aligned by a certified prosthetist. Participants were assigned a fitting and training order for the two conditions, either Locked-then-Flexible or Flexible-then-Locked. These assignments were made on an alternating basis (meaning if one participant was assigned Locked-then-Flexible, the next participant would be assigned Flexible-then-Locked) to avoid introducing a potential bias by fitting all participants in the same configuration. The alignment and fitting process was performed once by the prosthetist, held constant for all training and testing sessions, and kept the same for both foot configurations. After the fitting was complete, each participant was asked “On a scale from 1 to 10, with 10 being maximally satisfied, how satisfied are you with your alignment?” All participants rated their satisfaction with their experimental prosthesis alignment to be between 8 and 10 (mean: 9.4/10). At the end of the first training session, participants walked on the level treadmill in the Locked and Flexible toe joint configurations (for 5–10 min each) to begin acclimating to these feet.

During the second training session, the participants trained on stairs, on level and uneven terrain overground, and on a sloped treadmill. Each task was performed with both the Locked and Flexible configurations, using the assigned order from the first training day. In total, participants spent approximately 20 min walking on and acclimating to each foot configuration. Afterwards, participants were asked if they were satisfied with their training volume on the experimental prostheses. All participants reported scores of 9 or 10 on a 1–10 scale with 10 being fully satisfied.

At the conclusion of the second training session, participants were asked to rank their preferences for Foot One versus Foot Two. The investigator verbally asked the participant: “For level walking, did you prefer Foot One or Foot Two?”, where One and Two refer to the order each participant was assigned to complete the Locked versus Flexible toe joint configurations.

### Testing sessions

All participants were required to fast for the three hours preceding the metabolic data collection that occurred in the third session. They were also asked to refrain from consuming caffeine and from performing strenuous physical activity/exercising on the day of testing. When participants arrived at the laboratory, retro-reflective markers were affixed to their pelvis (4–6), thighs (8), knees (4), and shanks (8). On their intact limb, markers were also applied to the calcaneus (3) and metatarsal heads (2), and on their prosthetic limb, markers were applied to the cosmesis (3) and either side (3) of the prosthetic toe joint (6 total). During all data collection, three-dimensional motion capture (200 Hz; 10-camera system, Vicon, Oxford, UK) and synchronized ground reaction forces (1000 Hz; split-belt instrumented treadmill, Bertec, Columbus, USA) were collected. Level walking trials were a minimum of 5 min long, however, only 60-s of kinematic and kinetic data (cropped at random) advanced to the data processing stage. The inspired volume of oxygen (VO_2_) was continuously sampled (breath by breath) for the entire five-minute trial, using a portable metabolic system (COSMED K4b2, Rome, Italy). To aid in interpreting metabolic results we elected to complete level walking trials using a withdrawal study design (i.e., A-B-A design in a Flexible-Locked-Flexible order). In line with previous studies on similar populations^15,16^, participants walked on the treadmill at a constant speed of 1.14 ms^−1^.

We note that stair ascent and descent data were also collected in this session (after level walking), and uneven terrain and sloped walking were collected in a separate testing session. Specific methods related to these additional tasks are not detailed because only level walking data are presented and discussed in this manuscript.

### Data processing and analysis

Marker trajectories and ground reaction force data were low-pass filtered at 8 Hz and 15 Hz, respectively, with a fourth order Butterworth filter. Spatiotemporal variables (stride length and time; stance and swing time of each limb) were computed using the ground reaction force and foot/prosthesis marker data. Stride length was then non-dimensionalized by leg length ($L$) and all time variables were non-dimensionalized by $\sqrt{L/g}$, where $g$ is acceleration due to gravity^17,18^. Sagittal plane joint angles and net moments, net joint powers (six degree-of-freedom) and work, and center of mass dynamics (individual limbs method^19^) were computed in Visual3D (C-motion, Germantown, USA) and further processed using custom-built MATLAB (MathWorks, Natick, USA) functions. Moments and work were non-dimensionalized by $MgL$ where $M$ is body mass. Power was non-dimensionalized by $Mg\sqrt{gL}$ ^17,18^. Prosthesis power and work were also calculated in accordance with Takahashi and Stanhope^20^ and Zelik and Honert^21^. Center of mass work and prosthesis work were cropped to the Push-Off phase of gait using the positive range of center of mass power near terminal stance. Gross rate of oxygen consumption was estimated by averaging the last minute of raw VO_2_/min data per toe joint configuration and normalizing by body mass. Where relevant, group results are presented below as mean ± standard deviation. Average non-dimensionalization constants were 0.96 m (length), 0.31 s (time), 889.3 Nm/J (moment/work), and 2839.1 W (power). Some outcomes were redimensionalized for reporting purposes using these constants.

Maximum prosthetic toe joint angle was used to confirm the modified device was functioning as intended (i.e., reaching a significantly greater peak flexion angle in the Flexible versus Locked configuration). Spatiotemporal parameters were assessed to determine if any notable adjustments to these basic gait outcomes were present. Consistent with previous literature assessing assistive technology device design, we also compared prosthesis and center of mass Push-Off work, and gross rate of oxygen consumption between the two configurations^4,13,22,23^. Passive prostheses have been noted to contribute substantially less positive power during the Push-Off phase of gait, relative to the intact ankle joint^24^. This loss of power has also been observed at the center of mass level^25^. Restoring positive power during Push-Off may lead to improved metabolic cost^26^, ultimately reducing the muscular exertion required to walk using a passive prosthesis.

### Statistical analyses

All data were determined, via one sample Kolmogorov–Smirnov tests, to be non-normally distributed. Therefore, non-parametric repeated measures tests were used to assess differences between the Locked versus Flexible toe joints. Wilcoxon tests were used to investigate differences in all biomechanical variables. For gross rate of oxygen consumption, however, a Friedman test was used to compare the Flexible-Locked-Flexible (A-B-A) trials. Following this, Holm-Bonferroni corrections were applied to account for familywise error rates across the groups of principal kinematic/kinetic and spatiotemporal variables. For maximum toe joint angle, distal segment work and center of mass work the adjusted alpha levels were 0.025, 0.017 and 0.050, respectively. For intact limb stance time, prosthetic limb stance time, intact limb swing time, prosthetic limb swing time, stride time and stride length the adjusted alpha levels were 0.017, 0.025, 0.050, 0.008, 0.010 and 0.013, respectively. For gross rate of oxygen consumption an alpha level of 0.05 was applied. Statistical tests were conducted in MATLAB (MathWorks, Natick, USA).

## Results

### Spatiotemporal

Mean stride lengths were similar for Locked versus Flexible configurations (1.21 versus 1.20 m, respectively; p = 0.054; Table 2). Mean temporal variables (stride, stance, and swing times) were all within 0.02 s of each other for Locked versus Flexible configurations. These differences did not reach the adjusted threshold for significance when the Holm-Bonferroni method was applied, with the exception of prosthetic limb swing time (p = 0.008).

**Table 2:** Redimensionalized spatiotemporal variables.

	Stride length (m)	Stride time (s)	Prosthetic limb		Intact limb	
			Stance time (s)	Swing time (s)	Stance time (s)	Swing time (s)
Flexible (mean ± SD)	1.21 ± 0.08	1.07 ± 0.07*	0.67 ± 0.05	0.38 ± 0.04*	0.71 ± 0.05	0.34 ± 0.03
Locked (mean ± SD)	1.20 ± 0.08	1.06 ± 0.07*	0.67 ± 0.05	0.40 ± 0.04*	0.72 ± 0.05	0.35 ± 0.03

*Significant difference between conditions (p < 0.05). Note, statistical analyses were performed on dimensionless values.

### Joint kinematics

**Figure 2:**
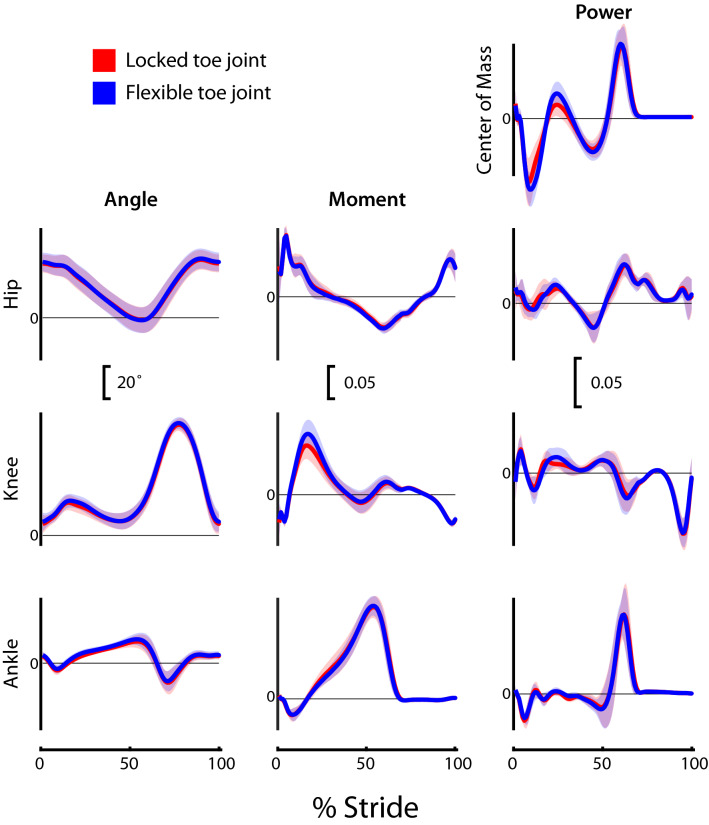
Intact limb (non-prosthetic) joint and center of mass dynamics for participants (*N* = 9) walking in a passive prosthesis with (i) a Flexible (dark blue line) and (ii) a Locked (light red line) toe joint configuration. Kinematic (angles) and kinetic (moments, powers) data were cropped into strides using ipsilateral heel strikes of the intact limb. Data presented as mean ± standard deviation (shaded regions). Using group mean re-dimensionalization constants, 0.05 corresponds to 0.47 Nm kg^−1^ for moments, and 1.50 W kg^−1^ for powers.

**Figure 3:**
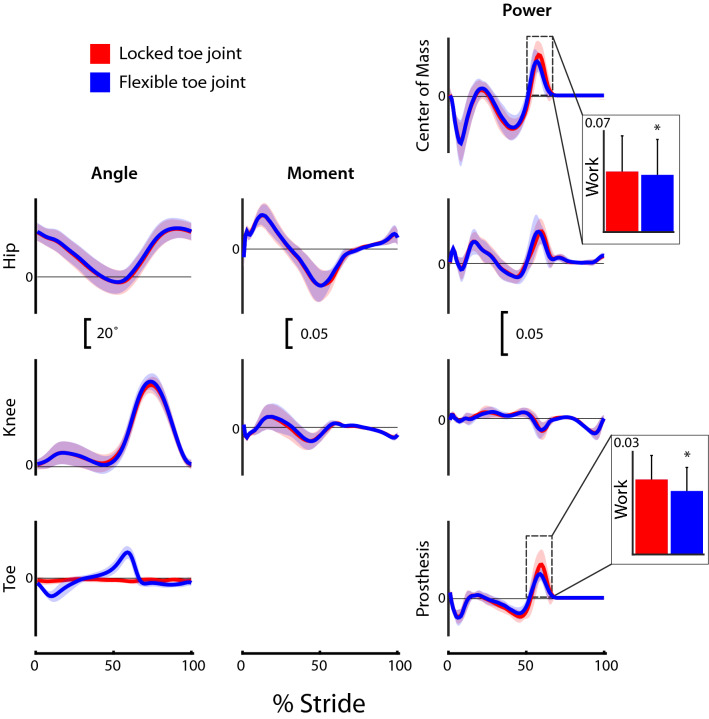
Prosthetic limb joint, center of mass and prosthesis dynamics for participants (*N* = 9) walking in a passive prosthesis with (i) a Flexible (dark blue line) and (ii) a Locked (light red line) toe joint configuration. Kinematic (angles) and kinetic (moments, powers) data were cropped into strides using ipsilateral heel strikes of the prosthetic limb. Positive prosthesis work and center of mass work were computed during Push-Off phase only (defined by center of mass power traces). Data are presented as mean ± standard deviation (shaded regions). Using group mean re-dimensionalization constants, 0.05 corresponds to 0.47 Nm kg^−1^/J kg^−1^ for moments and work, and 1.50 W kg^−1^ for powers.

Intact limb (non-prosthesis) ankle, knee, and hip angles, and prosthetic limb toe, knee, and hip angles are presented in Figs. 2 and 3, respectively. The average kinematic profiles at each joint were similar between configurations, with the exception of prosthesis toe joint angle. We observed a significant increase in maximum angle from 1.1 ± 1.6° in the Locked configuration, to 20.8 ± 3.0° in the Flexible configuration (p = 0.008), thus confirming our experimental design was effective in varying toe joint articulation.

### Joint kinetics

Most kinetic profiles were unaltered by the transition between Locked and Flexible toe joint configuration (Figs. 2 and 3). In fact, for all but the intact limb knee moment, prosthesis power, and center of mass powers, the group mean plots were visually indistinguishable. Positive prosthesis work during Push-Off was reduced in the Flexible configuration (0.018 ± 0.007 dimensionless, or 16.0 J) compared to the Locked configuration (0.021 ± 0.007 dimensionless, or 18.7 J; p = 0.004; Fig. 3). On average this decrease in prosthesis Push-Off work was 16% but ranged from 3 to 31% across our participant sample. The reduction in Push-Off also appeared in whole-body center of mass power (0.037 ± 0.023 dimensionless in the Flexible configuration versus 0.040 ± 0.023 dimensionless in the Locked configuration; p = 0.039; Fig. 3).

### Gross rate of oxygen consumption

**Figure 4:**
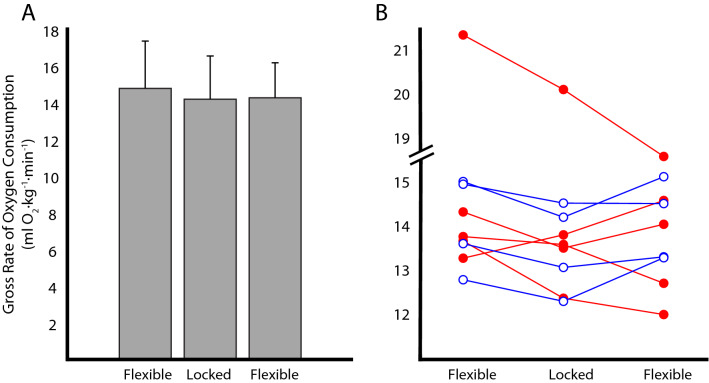
Effect of toe joint on gross rate of oxygen consumption. Results shown are from the A-B-A (Flexible-Locked-Flexible) study design. (**A**) Group means (*N* = 9) with error bars representing standard deviations. (**B**) Individual participant data points are indicated by variations in marker shape and color. Filled markers (red) indicate the user preferred the Flexible toe joint configuration and empty markers (blue) indicate the user preferred the Locked toe joint configuration.

No significant differences in gross rate of oxygen consumption were found between the Flexible-Locked-Flexible trials (p = 0.097; Fig. 4A). The initial Flexible test incurred 14.8 ± 2.6 ml O_2_ kg^−1^ min^−1^, the Locked configuration incurred 14.2 ± 2.4 ml O_2_ kg^−1^ min^−1^ and the final Flexible test incurred 14.3 ± 1.9 ml O_2_ kg^−1^ min^−1^. Subject-specific metabolic results are shown in Fig. 4B.

### User preference

Five users preferred the Locked configuration, while the remaining four preferred the Flexible configuration during level walking (Table 3).

**Table 3:** Participant preference, side of limb loss and self-identified limb dominance before amputation (if applicable).

Participant ID	User preference	Side of limb loss	Was the amputated limb dominant or non-dominant?
1	Flexible	R	Dominant
3		R	Dominant
4		R	Dominant
5		R	Dominant
2	Locked	L	Non-dominant
7		L	Non-dominant
8		L	Non-Dominant
9		L	N/A (ambipedal)
6		L	N/A (congenital)

We also reviewed subject-specific kinematics, kinetics, and metabolic results. We did not observe any signals, features or differences that seemed to explain or elucidate individual preferences with respect to Locked versus Flexible configurations. For brevity, subject-specific results are not presented here, but data/results are publicly archived.

## Discussion

Lower limb prosthesis users walking with the Flexible toe joint exhibited a decrease in Push-Off work: approximately 16% less from the prosthesis and 10% less at the center of mass level. Participants displayed little change in other joint kinematics or kinetics, and no apparent difference in rate of oxygen consumption versus the Locked toe joint configuration. Preferences were divided; four of the nine participants preferred walking with the Flexible toe joint, while the remaining five preferred the Locked toe joint. None of the traditional biomechanical or metabolic outcomes seemed to explain this observation. Interestingly, every participant who had an amputation on their dominant limb (defined below) preferred the Flexible toe joint, and all other participants preferred the Locked toe joint.

Kinematic and kinetic profiles from all intact and prosthetic limb joints were remarkably similar in the Flexible and Locked toe joint configurations (Figs. 2 and 3). This finding is consistent with previous results obtained when able-bodied persons walked with and without a toe joint using prosthetic adaptors^4^. Our observation that prosthesis Push-Off work decreased with the addition of the Flexible toe joint is also consistent with Honert et al.^4^. As noted by the authors^4^, reduced Push-Off was likely the result of the decreased effective length of the foot segment^27–29^.

We observed inconsistent changes in rate of oxygen consumption between the Locked and Flexible toe joint configurations (Fig. 4). Three participants appeared to have a monotonically decreasing trend across the A-B-A trials, one participant had a monotonically increasing trend, and four appeared to have a slightly lower oxygen consumption rate in the Locked (B) versus Flexible (A) configuration (Fig. 4B). The reason for this variability is unclear to us. No significant differences were detected at the group level. This may be explained by the small sample size or could be, in part, due to measurement limitations. For instance, prior studies report a minimum detectable change threshold of 0.8–1.0 ml O_2_ kg^−1^ min^−1^ associated with the metabolic equipment used^30,31^, and most participants in our study exhibited changes below this threshold. Nevertheless, across the A-B-A trials about half of the participants exhibited clear, reversible trends in oxygen consumption (i.e., a small decrease from A to B, followed a similar magnitude increase from B to A). This return to baseline suggests that the measurement resolution may have been sufficient for these participants, or at least better than the thresholds previously reported in literature; though again it is not clear why this observation held for some participants and not others. The biggest confound to the group level analysis was likely participants who exhibited monotonically increasing or decreasing trends in oxygen consumption over the A-B-A trials. Our study supports the conclusions of Lamers et al.^14^ who recently discussed similar challenges in interpreting group level statistical comparisons for a population of transtibial prosthesis users. Taken together, these findings highlight the benefits of using single-subject designs (including A-B-A protocols and subject-specific analysis methods) to gain more reliable insight regarding the effects of prosthetic interventions. Thus, the lack of statistical significance at the group level (particularly in small samples) should not automatically be interpreted to mean that certain individuals did not experience real, meaningful effects from an intervention.

All participants exhibited a higher magnitude of Push-Off work in the Locked configuration, but we cannot infer how this would be expected to have affected participants’ rate of oxygen consumption. This is because the relationship between device Push-Off work and metabolic cost is difficult to discern from the existing literature. For example, Caputo and Collins^26^ observed a significant reduction in metabolic cost when prosthesis Push-Off work was systematically increased; however, these findings were not replicated in a later study by the same group^32^. The onset of positive prosthesis Push-Off power is also likely to affect metabolic cost^33^. A noteworthy observation of the current study is that the user preference for seven of our nine participants did not correspond to the configuration that returned the lowest rate of oxygen consumption (Fig. 4B), with one possible explanation being that metabolic cost minimization was not the highest priority of our participant sample.

An unexpected surprise came as we compiled user demographic tables and noticed that all participants with right side limb loss preferred the Flexible toe joint (*N* = 4), while the remaining participants with left side limb loss preferred the Locked toe configuration (*N* = 5; Table 3). Given our relatively small sample size, this observation may be purely coincidental. However, the right versus left split perfectly matched the user preference results leading us to question if this phenomenon could be related to laterality (limb dominance). While there remains some debate about the roles of dominant and non-dominant lower limbs^34^, coordinated bilateral movement tasks (e.g., kicking a soccer ball) appear to rely on the dominant limb to execute the more dynamic aspect of the motion, with the non-dominant limb assuming a stabilizing role^34–36^. It therefore seems plausible that adding an additional degree-of-freedom into the foot keel might be preferred on the dominant limb, yet non-preferred on the non-dominant limb.

To explore this possibility, we followed up with each participant in this study to inquire about whether their right or left limb was dominant prior to amputation. One individual had congenital limb loss, which made the concept of limb dominance somewhat ill-defined, and as such we were unsure how to ask or establish which of their limbs was dominant. For the remaining eight non-congenital participants, we asked them to self-identify their dominant limb prior to amputation; specifically, we asked: “Before losing your leg, would you prefer to kick a soccer ball with your right or left leg?” Seven of eight individuals confidently identified as right-limb dominant, and one identified as ambipedal (non-discriminant). We compiled participant responses into Table 3, and the results were quite striking: all participants who had dominant limb loss preferred the Flexible toe joint, and all other participants preferred the Locked toe joint. This offers an intriguing avenue for future research to explore whether prosthesis users who have a dominant limb amputation exhibit different functional outcomes or device preferences than those who have a non-dominant limb amputation. If so, these findings could have important implications to prosthetic foot design and clinical prescription. Moving forward, we plan to collect limb dominance information from all prosthesis study participants, along with the other conventional demographic data such as height, weight, prescribed prosthesis, etc. We encourage other researchers in the field to record and report limb dominance as well. It seems likely that trends and insights may emerge organically in the scientific literature if limb dominance is reported alongside standard demographics (e.g., Table 1).

The results and interpretation here are based on data from nine K3/K4 level, unilateral below-knee prosthesis users. Based on discussions with clinicians, study participants, other end-users and prosthesis manufacturers, we suspect that the addition of a toe joint may actually be of most benefit and interest to K2 level individuals, for whom replacing lost Push-Off power may not be as important as restoring other aspects of mobility. However, the multi-task protocol we performed in this study was determined to be too strenuous for most K2 level participants. An interesting follow-up study would be to explore the effect of adding a toe joint in a K2 population, during a reduced set of locomotor tasks. We also note that the study participants were not fully blinded to the prosthesis configurations. This was for two reasons: (i) the toe section of the prosthesis was open/visible to allow for motion capture marker tracking, and (ii) our participants were very perceptive and, in general, they quickly felt the difference in toe/keel stiffness between the two configurations. To help mitigate biasing participants based on our language as experimenters, we referred to Locked and Flexible configurations as Foot One and Foot Two throughout data collections, and when asking for user preference.

In conclusion, the addition of a toe joint to a passive foot prosthesis reduced Push-Off work in all participants by approximately 2.5 J but appeared to have little effect on joint kinematics, kinetics, or rate of oxygen consumption. Participant preference for the Flexible or Locked toe joint during level walking was divided among our sample. The most intriguing, albeit preliminary, observation was the potential link between user preference and limb dominance—an area requiring further investigation.
